# Enterotoxigenic Escherichia coli Heat-Stable Toxin Increases the Rate of Zinc Release from Metallothionein and Is a Zinc- and Iron-Binding Peptide

**DOI:** 10.1128/mSphere.00146-20

**Published:** 2020-04-01

**Authors:** Mallory C. Kiefer, Natalya I. Motyka, John D. Clements, Jacob P. Bitoun

**Affiliations:** a Department of Microbiology and Immunology, Tulane University School of Medicine, New Orleans, Louisiana, USA; University of Kentucky

**Keywords:** *Escherichia* toxins, enteric pathogens, iron regulation

## Abstract

Enterotoxigenic Escherichia coli (ETEC) is a major diarrheal pathogen in children in low- to middle-income countries. Previous studies have identified heat-stable enterotoxin (ST)-producing ETEC as one of the major diarrhea-causing pathogens in children younger than five years. In this study, we examined iron and zinc binding by both human and porcine ST variants and determined how host metallothionein could detoxify ST. We found that ST purified from ETEC culture supernatants eluted as a doublet during C_18_ reverse-phase chromatography. Leading edge fractions of the ST doublet were found to be devoid of iron, while trailing edge fractions of the ST doublet were found to contain measurable iron. Next, we found that purified ST could be reconstituted with iron under reducing and anaerobic conditions, and iron-bound ST attenuated the induction of cGMP in T84 epithelial cells. Moreover, we demonstrated that supernatants of ETEC 214-4 grown under increasing iron concentrations were only able to induce cGMP at iron concentrations greater than 5 μM. *In vitro* studies also demonstrated that ST binds zinc, and once bound, zinc removal from ST required denaturing conditions. Zinc-bound ST also failed to induce cGMP. We found that ST contributes disulfide bonds to the perceived oxidized glutathione pool, increases the rate of zinc release from metallothionein, and can be detoxified by metallothionein. Lastly, we showed ST induces transcriptional changes in genes previously shown to be regulated by deferoxamine. These studies demonstrate ST ETEC pathogenesis may be tied intimately to host mucosal metal status.

**IMPORTANCE** Enterotoxigenic Escherichia coli (ETEC) is a major diarrheal pathogen in children in low- to middle-income countries, deployed military personnel, and travelers to regions of endemicity. The heat-stable toxin (ST) is a small nonimmunogenic secreted peptide with 3 disulfide bonds. It has been appreciated that dietary disulfides modulate intestinal redox potential and that ST could be detoxified using exogenous reductants. Using biochemical and spectroscopic approaches, we demonstrated that ST can separately bind iron and zinc under reducing conditions, thereby reducing ST toxicity. Moreover, we demonstrated that ST modulates the glutathione (GSH)/oxidized glutathione (GSSG) ratio and that ST should be considered a toxin oxidant. ST can be detoxified by oxidizing zinc-loaded metallothionine, causing free zinc to be released. These studies help lay a foundation to understand how diarrheal pathogens modulate intestinal redox potential and may impact how we design therapeutics and/or vaccines for the pathogens that produce them.

## Abstract (precis)

Enterotoxigenic Escherichia coli (ETEC) is a major diarrheal pathogen in children in low- to middle-income countries, deployed military personnel, and travelers to regions of endemicity. The heat-stable toxin (ST) is a small nonimmunogenic secreted peptide with 3 disulfide bonds. It has been appreciated that dietary disulfides modulate intestinal redox potential and that ST could be detoxified using exogenous reductants. Using biochemical and spectroscopic approaches, we demonstrated that ST can separately bind iron and zinc under reducing conditions, thereby reducing ST toxicity. Moreover, we demonstrated that ST modulates the glutathione (GSH)/oxidized glutathione (GSSG) ratio and that ST should be considered a toxin oxidant. ST can be detoxified by oxidizing zinc-loaded metallothionine, causing free zinc to be released. These studies help lay a foundation to understand how diarrheal pathogens modulate intestinal redox potential and may impact how we design therapeutics and/or vaccines for the pathogens that produce them.

## INTRODUCTION

Enterotoxigenic Escherichia coli (ETEC) is a significant global health threat. Most ETEC cases occur in children less than 5 years old in low- to middle-income countries (LMIC) and in travelers to LMIC (1, 2). The ETEC pathovar is defined by the production of the heat-labile enterotoxin (LT) and/or the heat-stable enterotoxin (ST). ETECs are classified as LT only, ST only, or LT and ST double positive. ETECs producing any combination of these toxins can cause secretory diarrhea in humans (3); however, ST-producing ETECs are one of the top four pathogens in children aged 0 to 60 months with moderate to severe diarrhea (MSD) (4, 5) and one of the top two pathogens in children aged 0 to 60 months with less severe diarrhea (LSD) (6). Despite progress, there are currently no licensed ETEC vaccines. Diarrheal illness from ETEC is self-limiting if rehydration is started early. WHO guidelines recommend zinc supplementation and oral rehydration therapy at the onset of ETEC- and enteric pathogen-mediated diarrheal illness (7, 8). Despite waning mortality from illness due to ETEC over the past decade, ETEC-attributable morbidity, including physical and intellectual stunting, continues to rise (9, 10).

STs are small nonimmunogenic peptides. There are at least two ST isoforms, namely, STh (human variant, 19 amino acids, NSSNYCCELCCNPACTGCY) and STp (porcine variant, 18 amino acids, NFTYCCELCCNPACAGCY). The heat stability of ST is a function of its compact size (∼2 kDa) and 6 cysteine residues forming three disulfide bonds (11). ST is a conformational mimic of the host natriuretic peptide hormones guanylin and uroguanylin (12), which regulate salt and water movement over renal and intestinal epithelia. Uroguanylin has at least two topoisomers called uroguanylin A and uroguanylin B, which can be differentiated based on the peptide orientation when the final disulfide bond is covalently coordinated. Uroguanylin A potently activates the intestinal guanylyl cyclase C (GC-C) receptor, and although uroguanylin B weakly activates intestinal GC-C, it has high natriuretic activity in the kidney (13, 14). The GC-C receptor is a single transmembrane receptor with an extracellular domain (15), a transmembrane domain, a kinase domain, a hinge region, and a guanylate cyclase catalytic domain (16). Upon ST binding, the GC-C receptor is internalized (17) and a signal is transduced to the guanylate cyclase catalytic domain, resulting in increased intracellular cGMP (18, 19). Accumulation of intracellular cGMP results in the opening of cystic fibrosis transmembrane conductance regulator (CFTR) through three different signaling pathways in intestinal epithelial cells: (i) direct activation of protein kinase G II (8, 20), (ii) direct activation of protein kinase A (PKA) (21), and (iii) inhibition of phosphodiesterase (PDE) 3, indirectly activating PKA (18). Besides opening CFTR, increased intracellular cGMP inhibits the sodium/hydrogen exchange channel (NHE3) on the luminal surface of intestinal epithelial cells and blocks sodium absorption from the intestinal lumen (16, 18, 20). Altogether, ST induces accumulation of salt ions in the intestinal lumen, and water follows osmotically, resulting in the clinical diagnosis of secretory diarrhea.

Enteric pathogens are exposed to incessant fluctuations in environmental conditions, including nutrient and oxygen availability, microbiome metabolites, mucus, and host defense systems. For enteric pathogens, transition metal availability signals the arrival to a potential colonization niche, and they have developed sophisticated mechanisms to acquire iron and zinc for bacterial metabolism. Transition metal availability regulates the production of Shiga toxin (22) of *Shigella* and siderophore and diphtheria toxin production in Corynebacterium diphtheriae (23). Enteric pathogens secrete high-affinity siderophores/zincophores, including aerobactin, enterobactin, and yersiniabactin, to scavenge iron/zinc from the environment (24, 25) for pathogenesis. Excess iron represses the expression of colonization factor antigen I (CFA/I) fimbriae in ETEC type strain H10407 via iron-sulfur cluster regulator protein, IscR (26, 27). IscR and iron mediate virulence in Shigella flexneri, Erwinia chrysanthemi, and Pseudomonas aeruginosa (28–30). Zinc induces the production of LT in some ETEC isolates (31), and zinc supplementation does not generally impact ETEC viability or adhesion to host epithelia yet alters the transcriptome of ETEC and the host (32, 33).

There is a gap in knowledge regarding the importance of luminal redox potential, transition metal availability, and microbial by-products on active enteric infections in the intestinal lumen. Recently, single-cell RNA sequencing provided a deeper glimpse into the subsets of highly specialized intestinal epithelial cells from colonic biopsy specimens of patients (34). One such cell type, called BEST4OTOP2, expresses proteins to regulate luminal pH, secretes both uroguanylin and guanylin, and expresses genes of the metallothionein family, contributing to free radical defense and metal ion storage and transport (34). Since ST is a mimic of uroguanylin and guanylin, it is possible that ST also functions on this cell type or neighboring epithelial cells. Moreover, Rivera-Chavez and Mekalanos have recently shown that iron acquisition and vibriobactin siderophore genes confer a growth advantage to Vibrio cholerae only when cholera toxin is produced (35), positioning enterotoxins as moonlighters for pathogen-mediated acquisition of host nutrients. In support of this, Rivera-Chavez and Mekalanos have also shown that cholera toxin induces the bioavailability of heme, long-chained fatty acids, and l-lactate in the terminal ileum (35).

Recent RNA-seq data of stool samples from an ETEC human infection challenge model suggests that anaerobiosis is central to modulating ETEC virulence (36). Classical ETEC virulence factors, including CFA/I (encoded by the *cfaABCDE* operon) and LT (encoded by *eltAB*) were shown to be downregulated during anaerobiosis by the iron-sulfur cluster containing the oxygen-sensitive fumarate and nitrate reduction (FNR) regulator (36). Iron and zinc are transition metals essential for nearly all organisms, and both hosts and pathogens have developed sophisticated mechanisms to acquire and safeguard them (37). Interestingly, the WHO recommends zinc supplementation in addition to oral rehydration therapy at the onset of diarrheal illness. In the host, zinc helps maintains the redox potential of glutathione (38) and induces expression of antioxidant and zinc-binding protein metallothionein (39). Moreover, zinc-deficient diets that allow ETEC H10407 and other enteric pathogens to colonize the murine intestinal epithelium have recently been developed (40–42), and iron limitation has been used to induce expression of the colonization factor fimbrial subunit CfaB in ETEC H10407 through the iron-sulfur cluster regulator (IscR) (26).

The unique sequence of ST, bearing 3 disulfide bonds, suggests that ST ETEC may be able to modulate the luminal oxidant pool, via ST presentation adding disulfide bonds to the oxidized glutathione pool. Moreover, after reduction, the cysteine residues of ST could bind zinc or iron and lead to further ST detoxification. In this study, we sought to understand the impact of iron and zinc on the ability of ST to induce cGMP in T84 epithelial cells, a proxy for ST secretory activity, and to investigate the role of metallothioneins in ST detoxification.

## RESULTS

### Purification of iron-bound ST from ETEC supernatants.

**FIG 1:**
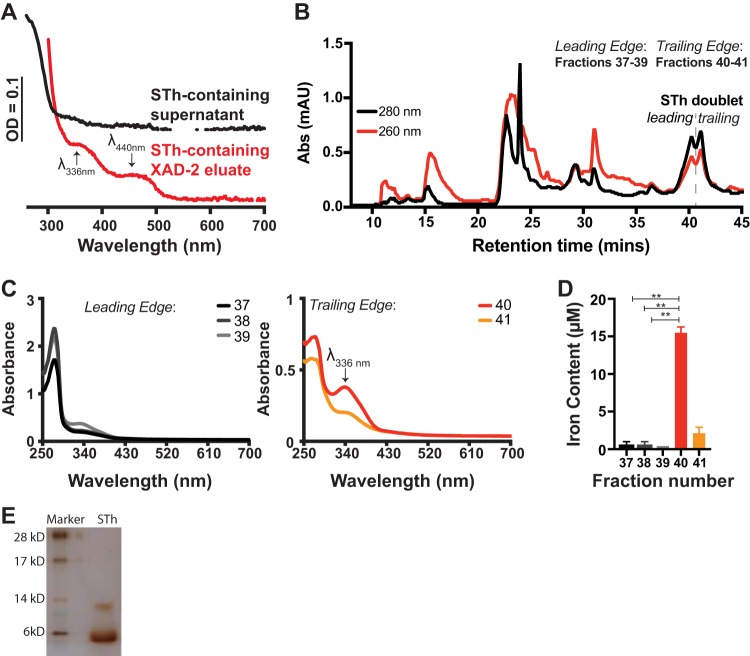
Heat-stable enterotoxin (ST) is purified with iron bound. (A) UV-Visible spectra of STh-containing supernatant (black trace) and XAD-2 column eluate (red trace) showing unique spectral peaks/shoulders at 340 nm and 440 nm from ETEC 9115 fermentation. (B) Representative elution profile of the final step in ST purification. ST-containing material was applied in ammonium acetate, pH 5.6, in water, and ST was eluted with 50% methanol using an isocratic flow of ammonium acetate, pH 5.6, in methanol as the mobile phase. As verified by cGMP accumulation in T84 epithelia, the majority of STh elutes as a doublet near 40 min of retention time and corresponds to fractions 37 to 41. (C) UV-Visible spectra of the leading edge (left, fractions 37 to 39) and trailing edge (right, fractions 40 to 41) of the STh doublet. The UV-Visible spectra of trailing-edge fractions 40 and 41 display the same maximum wavelength (λ_max_) at 336 nm as shown in panel A. Also, in trailing-edge fractions, the ratio of the peak height at 336 nm to the peak height at 280 nm suggests iron binding to ST at a higher occupancy than in leading-edge fractions. The data presented in panels A to C are representative of at least three biological replicates. (D) Iron quantification with Ferrozine/cysteine of fractions 37 to 40, corresponding to the ST doublet, shows that trailing-edge fractions 40 and 41 contain more iron than leading-edge fractions 37 to 39. Values from iron binding analysis are from a representative purification run, assayed in triplicates, and values for are means ± standard deviations, when applicable. **, *P* < 0.005. (E) A silver-stained low-molecular-weight gel shows that purified STh migrates to approximately 6 kDa with a minor band at 12 kDa. Matrix-assisted laser desorption ionization–time of flight (MALDI-TOF) analysis of this ST preparation yielded a mass/charge ratio of 2,048.1 g/mol.

ETEC strain H10407 (NCBI taxonomy identifier [ID] 316401) is commonly used in human ETEC infection models (36, 43, 44) and produces LT, STp, and STh (3, 45). Each enterotoxin alone (LT, STh, and STp) is capable of causing secretory diarrhea (3). The presence of multiple enterotoxins in H10407 has led to the interest of ETEC challenge models using ST-only ETEC isolates which would be specifically suited to determine protective efficacy of ST toxoids or genetic ST fusions as immunogens (3, 11, 12, 46). When Evans and Evans first identified ETEC H10407, they noted that upon concentration of enterotoxin-containing supernatants by ammonium sulfate precipitation, tox^+^ strains were of a darker coloration than tox^−^ strains (47). When we routinely purify ST from ETEC culture supernatants, an early indicator that a particular ETEC strain makes functional ST is the reddish-purple color of bulk ST-containing supernatant (see Fig. S1 in the supplemental material). One explanation of the color that we see is the potential for iron-binding proteins to be secreted into the supernatant. Despite seeing the reddish-purple color in bulk supernatant, we do not see unique UV-visible (UV-Vis) spectral features of the dilute supernatant, indicating metal binding (Fig. 1A, black trace). However, after concentrating the ST-containing supernatant to a hydrophobic XAD-2 resin, we routinely see the reddish-purple color concentrate on the XAD-2 resin and the UV-Vis spectrum of the 10-fold-concentrated ST-containing XAD-2 eluate shows unique spectral peaks at 336 nm and 440 nm (Fig. 1A, red trace), indicative of iron binding. The ST-containing XAD-2 eluate is then applied to a Bio-Gel P6 column (data not shown), concentrated, and finally applied to a C_18_ column in reverse-phase high-performance liquid chromatography (HPLC). ST elutes from the C_18_ column in around 50% methanol, which is indicated by the dashed line and STh doublet (Fig. 1B) (48). As shown in this representative C_18_ elution profile, absorbance at 280 nm (black trace) is greater than the absorbance at 260 nm (red trace), indicating peptide concentration is greater than nucleic acid concentration, for an extended time at around 40 min retention time, indicating STh elution (doublet). Importantly, others have also seen that ST eluates as a doublet from hydrophobic columns (49) or that ST isoforms are eluted in multiple peaks (50). However, it is worth noting that we do not see doublet during purification of STp from supernatants of ETEC B41 (data not shown). Here, we refer to fractions 37 to 39 as ST doublet leading-edge fractions and fractions 40 to 41 as ST doublet trailing-edge fractions. When assessed via UV-Vis spectrophotometry, leading-edge fractions (37 to 39) show a dominant spectral peak at 280 nm (Fig. 1C, left), and trailing-edge fractions (40 and 41) have two discrete peaks, one at 280 nm and another at 336 nm (Fig. 1C, right), which is the same wavelength as the peak seen following elution of ST-containing material after XAD-2 chromatography (Fig. 1A, red trace). STh doublet fractions were the assessed for the presence of iron using the Ferrozine and l-cysteine method, as previously conducted (51). Iron quantification analysis showed that doublet trailing-edge fractions 40 to 41 (Fig. 1D) have iron bound. In fraction 40, the ratio of the absorbance of the peak at 336 nm to the absorbance of the peak at 276 nm is greater than 0.5, indicating an ST fraction capable of iron binding. Silver-stained low-molecular-weight gel electrophoresis shows the purity (Fig. 1E) of the pooled STh containing fractions used in following experiments.

10.1128/mSphere.00146-20.1 FIG S1 ST-containing supernatant of ETEC 9115 (STh overexpression strain) is purple-red in color. This crude material also induces cGMP when applied to T84 epithelial cells. Download FIG S1, EPS file, 1.9 MB. Copyright © 2020 Kiefer et al. 2020 Kiefer et al. This content is distributed under the terms of the Creative Commons Attribution 4.0 International license.

### Iron binding to STh and STp regulates the ability to induce cGMP in T84 cells.

**FIG 2:**
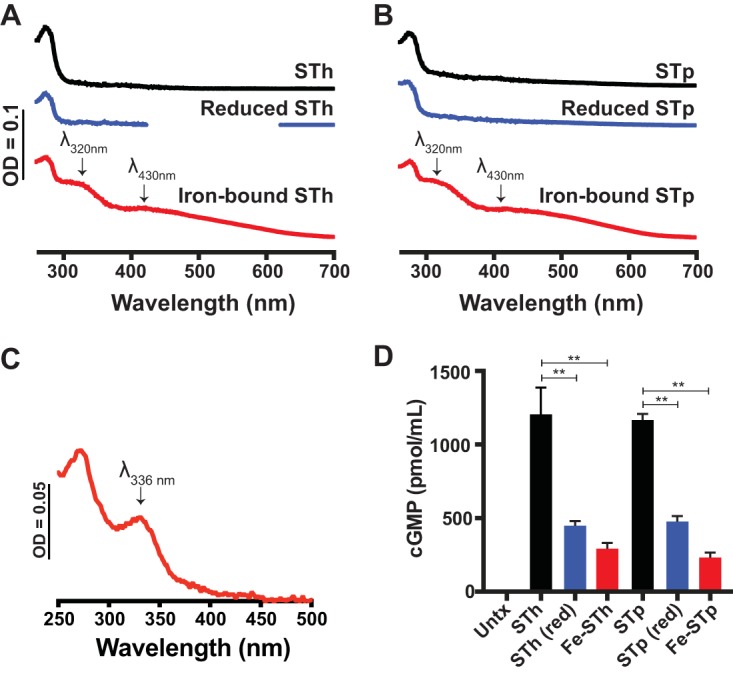
Reconstitution of ST with iron reduces its ability to induce cGMP in T84 cells. (A) UV-Visible spectra of 100 μM purified STh (black trace), DTT-reduced STh (blue trace), and anaerobically reconstituted STh in the presence of ferrous iron (red trace). The reaction mixtures were desalted to remove excess DTT and/or free iron. Spectral peaks at 430 nm and 315 nm are indicative of iron binding. (B) UV-Visible spectra of 100 μM purified STp (black trace), DTT-reduced STp (blue trace), and anaerobically reconstituted STp in the presence of ferrous iron (red trace). The contents of the reactions were desalted to remove excess DTT and/or free iron. Spectral peaks at 430 nm and 315 nm are indicative of iron binding. (C) UV-Visible spectra of anaerobically reconstituted STh exposed to atmospheric conditions for 18 h. The emergence of λ_max_ at 336 nm corresponds to the spectra we see with purified ST trailing-edge fractions in Fig. 1C. The data presented in panels A to C are representative of at least three biological replicates. (D) Iron binding to STh or STp reduces its ability to induce cGMP compared to that of native toxin in T84 cells. Values are means ± standard deviations versus ST alone. **, *P* < 0.005; Untx, untreated.

Recent studies have addressed the expression of ETEC virulence factors in response to anaerobiosis in the human host (36), but few, to our knowledge, have sought to understand the role of transition metals on ETEC enterotoxin virulence. Since we found measurable iron in ST purified directly from the supernatant of ETEC cultures, we next wanted to determine if purified STh and STp could be reconstituted more fully with iron *in vitro* and determine the impact of iron-bound ST on the induction of cGMP in target epithelia. Both purified STh (Fig. 2A) and STp (Fig. 2B) were capable of coordinating exogenously added iron under anaerobic conditions, as depicted by spectral peaks at 320 nm and 430 nm (red traces in Fig. 2A and B). Kinetics of iron binding to STh, as represented by the increase in absorbance at 430 nm, which has been used previously as a proxy for iron binding to protein (52, 53), shows that ST does not bind iron very well aerobically (see Fig. S2A and B). This suggests that iron binding to ST may be oxygen labile, as has been noted with other iron-binding enzymes, including aconitase and FNR (54). In an effort to understand the effect of atmospheric oxygen on iron-reconstituted STh, we exposed anaerobically reconstituted ST to air overnight. Exposing anaerobically reconstituted ST to atmospheric conditions (Fig. 2C) recapitulates the UV-Vis spectra of iron-bound ST typically seen in fraction 40 during ST purification (Fig. 1C) with a peak at 336 nm, suggesting oxidation of the iron center. Next, we applied equal masses of native, partially reduced, and iron-reconstituted ST to T84 cells to determine their abilities to induce cGMP. As shown in Fig. 2D, iron-reconstituted STh and STp are hindered in their abilities to induce cGMP in T84 cells, unlike native STh or STp, which both induce high levels of cGMP. Importantly, in the patent mouse model of toxin-mediated secretion, iron-reconstituted ST induced significantly less secretion than native ST (Fig. S2C).

10.1128/mSphere.00146-20.2 FIG S2 Kinetics of *in vitro* iron reconstitution of STh under anaerobic (A) and aerobic (B) conditions and patent mouse model. ST (100 μM) was incubated in the presence of DTT (2 mM) and ferrous ammonium sulfate (400 μM), either in a sealed cuvette purged with argon (A) or an open-to-air cuvette (B). Baseline spectra are represented proximal to 0-min labels, and then reconstitution was initiated and overlaid spectra were recorded every 3 min for 15 (A) or 30 (B) min. The arrows represent iron binding to ST and are quantified by the absorbance of the spectral peak at 430 nm, as seen in the anaerobic iron reconstitution reaction (A) but not in the aerobic reconstitution reaction (B). (C) Patent mouse model of ST enterotoxicity shows fluid accumulation in the intestinal lumen of female 6-week-old BALB/c animals (*n* = 3 per group) treated with native STh (25 μg) as opposed to animals treated with iron-reconstituted STh (25 μg). Download FIG S2, EPS file, 1.9 MB. Copyright © 2020 Kiefer et al. 2020 Kiefer et al. This content is distributed under the terms of the Creative Commons Attribution 4.0 International license.

### Low iron availability suppresses ST-mediated induction of cGMP in T84 cells.

**FIG 3:**
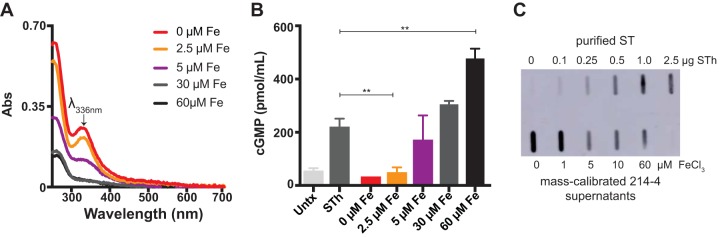
Iron availability in 4AA medium affects the ability of ST to stimulate cGMP in T84 cells. (A) UV-Visible spectra of supernatants of ETEC 214-4 (STp^+^ CS6^+^) grown in chemically defined medium with iron concentrations as indicated. At low iron concentrations (0 to 5 μM), the supernatants were reddish colored, indicated by λ_max_ at 336 nm. At higher iron concentrations (10 to 60 μM), the supernatants were not reddish and did not have a λ_max_ at 336 nm. The data presented are representative of at least three biological replicates. (B) Supernatants (10 μl) of ETEC 214-4 grown at different iron conditions were assessed for their ability to induce cGMP in T84 epithelia. As shown, at low iron concentrations (0 to 2.5 μM), when λ_max_ at 336 nm was present, ETEC 214-4 supernatants were unable to stimulate cGMP accumulation in T84 epithelia. When 5 μM iron was added, ETEC 214-4 supernatants appeared to reach a midpoint equilibrium of cGMP accumulation in T84 epithelia. At high iron concentrations (10 to 60 μM), when λ_max_ at 336 nm was absent, ETEC 214-4 supernatants were able to stimulate cGMP accumulation in T84 epithelia. For STh positive control, 5 ng of purified toxin was applied. Values are means ± standard deviations versus ST alone. **, *P* < 0.005. (C) Slot blot ETEC 214-4 supernatants after overnight culture in 4AA medium with different FeCl_3_ concentrations (bottom row) compared to a standard curve of ST (top row). Total protein content of the supernatants was calibrated to 15 μg before loading onto the slot blot. The membrane was loaded, blocked with 5% BSA for 1 h, and then incubated with protein A-purified anti-ST-KLH (1:400) overnight. The membrane was washed in PBS five times, and then anti-rabbit IgG conjugated to horseradish peroxidase (HRP) was added for 1 h, followed by five PBS washes and chemiluminescence detection with Pierce ECL substrate. Overall, there appeared to be more antigenic ST produced and secreted at low iron concentrations than at high iron concentrations. Untx, untreated.

In the presence of oxygen, free ferrous iron has the potential to participate in Fenton chemistry to generate deleterious free radicals and is therefore tightly regulated in biological systems. In an effort to determine if iron directly or indirectly regulates ST production or ST folding in ST^+^ ETEC isolates, we titrated iron ranging from 0 to 60 μM FeCl_3_ in the 4-amino-acid (4AA) chemically defined medium. We chose to assess the impact of iron on ST^+^ ETEC clinical isolates 214-4 (STp producer) (Fig. 3) and 504239 (STh producer) (see Fig. S3), since previous studies have demonstrated that supernatants from these strains induce robust and consistent levels of cGMP when applied to T84 cells (3, 55). In both ETEC 214-4 and 504239, iron availability during growth influenced the ability of supernatants from stationary phase to induce cGMP when applied to T84 cells (Fig. 3 and S3). When grown at low iron concentrations (0 to 2.5 μM), supernatants from ETEC 214-4 displayed the previously identified spectral peak at 336 nm (Fig. 3A) and were unable to induce cGMP when applied to T84 cells (Fig. 3B). On the other hand, when grown at high iron concentrations of >5.0 μM, supernatants from ETEC 214-4 lost the spectral peak at 336 nm (Fig. 3A) and induced cGMP when applied to T84 cells (Fig. 3B). It is worth noting that the iron concentration during ETEC fermentation for ST purification is 30 μM and would favor ST capable of inducing cGMP in T84 cells (Fig. 3B). Then, we applied 15 μg total protein from the supernatants of ETEC 214-4 grown at each iron concentration to a slot blot apparatus and probed for ST antigenicity using a protein A-purified anti ST-KLH polyclonal rabbit antibody. Surprisingly, no- to low-iron conditions (0 to 2.5 μM iron), where culture supernatants are unable to induce cGMP, showed the highest ST antigenicity (Fig. 3C), further supporting the data that ST is present but nontoxic at low-iron conditions. Overall, there appears to be more antigenic ST produced and secreted at low iron concentrations than at high iron concentrations. These data indicate that in certain ETEC strains, as iron increases, ST production/secretion decreases but ST toxicity increases (as measured by the ability of ST to induce cGMP in T84 cells), and the transition between inactive ST (iron bound) and active ST (disulfide laden) could be determined using the amplitude of the spectral peak at 336 nm of ST-containing supernatants.

10.1128/mSphere.00146-20.3 FIG S3 Iron availability during growth affects the ability of ST-containing supernatants from ETEC 504239 to stimulate cGMP in T84 cells. (Top) UV-Visible spectra of supernatants of ETEC isolate 504239 (STh^+^ CFA/I^+^) grown in chemically defined medium with iron concentrations as indicated. At low iron concentrations (0 to 5 μM), the supernatants were reddish colored, indicated by λ_max_ at 336 nm. At higher iron concentrations (10 to 60 μM), the supernatants were not reddish and did not have a λ_max_ at 336 nm. (Bottom) Supernatants (10 μl) of ETEC 504239 grown under different iron conditions were assessed for their ability to induce cGMP in T84 epithelia. As shown, at low iron concentrations (0 to 2.5 μM), when λ_max_ at 336 nm was present, ETEC 504239 supernatants were unable to stimulate cGMP accumulation in T84 epithelia. When 5 μM iron was added, ETEC 504239 supernatants appeared to reach a midpoint equilibrium of cGMP accumulation in T84 epithelia. At high iron concentrations (10 to 60 μM), when λ_max_ at 336 nm was absent, ETEC 504239 supernatants were able to stimulate cGMP accumulation in T84 epithelia. For STh positive control, 5 ng of purified toxin was applied. Untx, untreated. Download FIG S3, EPS file, 1.7 MB. Copyright © 2020 Kiefer et al. 2020 Kiefer et al. This content is distributed under the terms of the Creative Commons Attribution 4.0 International license.

### STh and STp are zinc-binding peptides.

**FIG 4:**
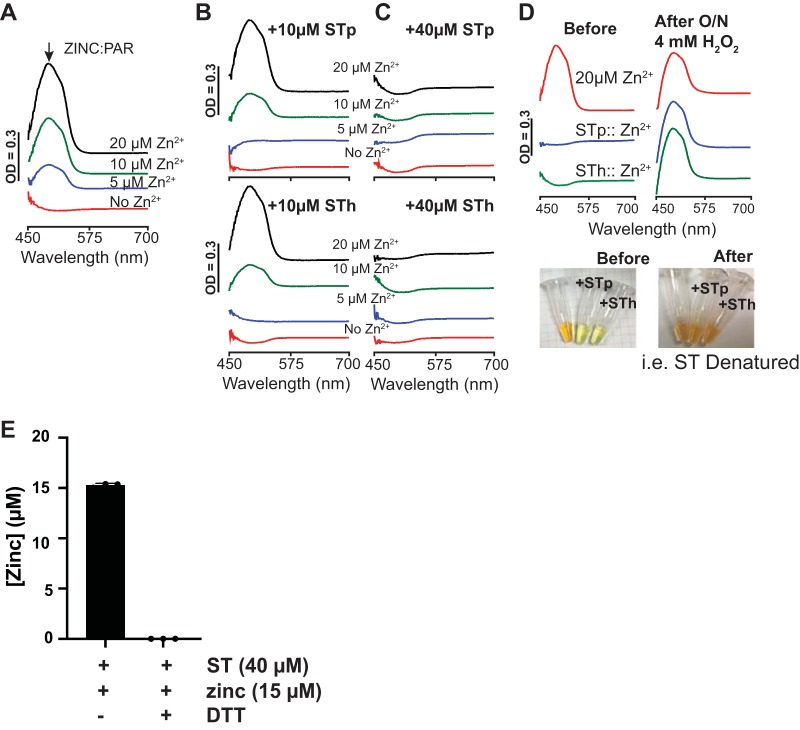
ST is a zinc-binding peptide. (A) Formation of the Zn:PAR_2_ complex can be quantified by the increase in absorbance at 490 nm as zinc concentration increases. (B) Addition of 10 μM STp to the reaction mixture used for the data in panel A inhibited the formation of the Zn:PAR_2_ complex when 5 μM zinc was added and decreased the amplitude of the peak at 490 nm (Zn:PAR_2_ complex) when 10 μM and 20 μM zinc were added. Addition of 40 μM STp to the reaction mixture used for the data in panel A inhibited the formation of the Zn:PAR_2_ complex when 5, 10, and 20 μM zinc were added. (C) Addition of 10 μM STh to the reaction mixture used for the data in panel A inhibited the formation of the Zn:PAR_2_ complex when 5 μM zinc was added and decreased the amplitude of the peak at 490 nm (Zn:PAR_2_ complex) when 10 μM and 20 μM zinc were added. Addition of 40 μM STh to the reaction mixture used for the data in panel A inhibited the formation of the Zn:PAR_2_ complex when 5, 10, and 20 μM zinc were added. (D) Zinc can be released from STp and STh by incubating overnight at room temperature with 4 mM H_2_O_2_. Once the peptides become denatured, PAR binds the zinc once bound by STp and STh, resulting in the formation of the peak at 490 nm (Zn:PAR_2_ complex). The data presented are representative of at least three replicates. (E) Zinc competition reaction between PAR and ST shows PAR:zinc complex formation when ST (40 μM) plus zinc (15 μM) was incubated for 20 min at room temperature without DTT (−) or with 2 mM DTT (+). ST outcompeted PAR for zinc binding under reducing conditions (+DTT, shown by the absence of the PAR:Zn complex), presumably based on free thiols.

The WHO recommends zinc supplementation in addition to oral rehydration therapy for 2 weeks at diarrheal disease onset, including diarrheagenic ETEC. Proteins rich in cysteine residues have a well-documented role in binding multiple transition metals, including metallothionein and calprotectin of the host. Since we found that ST binds iron, we set out to determine if ST also binds zinc based on the proximity of the conserved cysteines and to determine how zinc modulates the ability of ST to induce cGMP in T84 cells. To determine if ST binds zinc, we used the chemical chelator 4-(2-pyridylazo)resorcinol (PAR). Two molecules of PAR will bind one molecule of zinc to form a Zn:PAR_2_ complex that emits a quantifiable spectral peak at 490 nm under oxidative or reducing conditions. The concentrations of PAR and dithiothreitol (DTT) were fixed and zinc was titrated at increasing concentrations to show the dependency of zinc on the amplitude of the peak at 490 nm. As shown in Fig. 4A, incubation of PAR and free zinc caused Zn:PAR_2_ complex formation, with more Zn:PAR_2_ complex formation as the concentration of zinc increased from 0 μM (red trace) to 5 μM (blue trace), 10 μM (green trace), and 20 μM (black trace). In a subsequent reaction, we added either 10 μM STp or STh (Fig. 4B) with free zinc, PAR, and DTT, as in Fig. 4A As shown in Fig. 4B, when 5 μM zinc was added to reaction mixture containing PAR and ST, Zn:PAR_2_ complex formation was inhibited (compare the blue line in Fig. 4A to the blue line in Fig. 4B). At zinc concentrations of 10 μM (green lines) and 20 μM (black lines), Zn:PAR_2_ complex formation occured (Fig. 4B), albeit to a lesser amplitude than without ST (Fig. 4A). Next, we increased the STp and STh concentrations 4-fold to 40 μM and incubated them each (Fig. 4C) with free zinc, PAR, and DTT. At 40 μM ST, Zn:PAR_2_ complex formation was inhibited with up to 20 μM zinc added, suggesting that 40 μM ST is capable of binding all 20 μM zinc added (i.e., generation of the classic Zn:PAR_2_ peak at 490 nm was completely inhibited). Taken together, these data suggest that under these conditions, (i) the affinity between ST and zinc is tighter than the affinity between PAR and zinc and (ii) the ST and zinc binding ratio is 2 ST:1 Zn. For example, when 5 μM free zinc is added to 10 μM ST, we do not see Zn:PAR_2_ formation. Similarly, when 20 μM free zinc is added to 40 μM ST, we do not see Zn:PAR_2_ formation. However, when 10 μM free zinc is added to 10 μM ST, we do see Zn:PAR_2_ formation. This suggests that ST becomes saturated before PAR begins to bind zinc. Next, we sought to determine whether zinc could be released from ST once bound. For these studies, we incubated free zinc, zinc-bound STp, and zinc-bound STh with PAR. Of these three conditions, only free zinc and PAR incubation reaction mixtures showed the Zn:PAR_2_ complex at 490 nm (Fig. 4D, red trace). Denaturing conditions, including overnight incubation with 4 mM H_2_O_2_ (Fig. 4D) and boiling at 100°C for 30 min (data not shown), forced the release of zinc from ST and subsequent binding of zinc to PAR, as seen by the formation of the Zn:PAR_2_ complex at 490 nm (Fig. 4D). Computational analysis using ion ligand binding site prediction parameters software IonCom also predicted that ST is a high-affinity zinc-binding peptide (data not shown) (56). It is important to note that in order for ST to compete with PAR for zinc binding, ST must be reduced (Fig. 4E). ST can only compete with PAR for zinc binding under reducing conditions (plus DTT, shown by the absence of the PAR:Zn complex in Fig. 4E), presumably based on free thiols. Future studies will attempt to pinpoint the required cysteine residues for zinc binding.

### Zinc-bound ST does not induce cGMP in T84 cells and zinc chelation affects ST toxicity.

**FIG 5:**
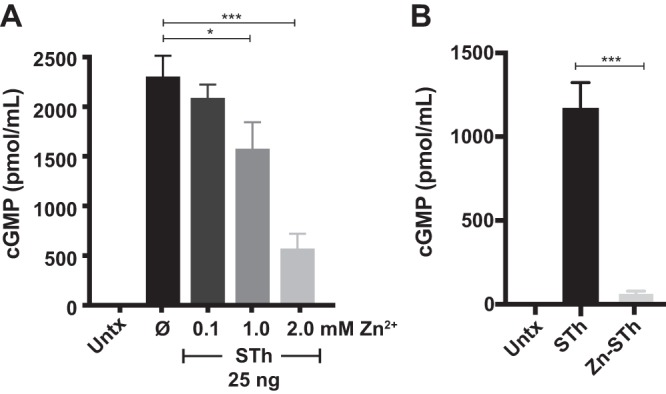
Zinc inhibits ST-mediated induction of cGMP in T84 epithelia. (A) Zinc (0.1 mM to 2.0 mM) was added to T84 cells 1 h prior to ST treatment with zardaverine (20 μM) and vardenafil (50 μM). At high zinc concentrations, ST-mediated induction of cGMP was significantly decreased. (B) *In vitro* reconstituted zinc-ST failed to induce cGMP accumulation in T84 epithelia compared to that with STh. Values are means ± standard deviations versus ST alone. *, *P* < 0.05; ***, *P* < 0.0005; Untx, untreated; Ø, no zinc added.

Next, we sought to determine the effect of zinc supplementation on ST-mediated induction of cGMP in T84 epithelia. Previously, it was reported that ST induced production of cGMP in Caco-2 cells regardless of zinc supplementation (57). However, using T84 epithelial cells, we demonstrated that exogenously added zinc hinders the ability of ST to induce cGMP (Fig. 5A), especially at concentrations of >1.0 mM. The difference in these findings could be a function of the amount or purity of ST used in the different assays. Our studies used up to 10-fold less ST than previously reported in Caco-2 epithelial cells (57). Moreover, quantitative PCR (qPCR) analysis demonstrated that GC-C expression is higher in T84 cells than in Caco-2 cells, making T84 cells a more reliable tissue culture model than Caco-2 cells for measuring ST activity (data not shown). It is possible that high zinc concentrations could be found in cases of zinc supplementation and oral rehydration therapy, since intestinal absorption of zinc is significantly aided by sugar (58). Kinetic studies show that the rate of small intestinal zinc absorption is proportional to the concentration of zinc perfused over the range of 0.1 to 3 mM zinc (58). Moreover, we show that preformed zinc-bound ST was unable to induce cGMP in T84 cells (Fig. 5B). The 4AA chemically defined medium used to induce ST expression from clinically relevant ST^+^ ETEC isolates is devoid of zinc. In an attempt to understand the impact that zinc supplementation would have on ST production from ST^+^ ETEC clinical isolates, we added 10 μM ZnSO_4_ to the growth medium. We grew clinically relevant ST^+^ ETEC isolates (3) side by side in zinc-replete and zinc-deplete media. After overnight growth, we harvested the supernatants and applied the same volumes of supernatants to T84 epithelia for assessment of ST production. Zinc supplementation during ST^+^ ETEC growth in minimal medium decreases the threshold of cGMP induction by ST^+^ ETEC supernatants (see Fig. S4). Future studies in different ST^+^ ETEC isolates will determine if zinc supplementation alters ST production/secretion or ST activity.

10.1128/mSphere.00146-20.4 FIG S4 Zinc supplementation into ETEC growth medium decreases the ability of supernatants to induce cGMP. ETEC clinical isolates 504838, 300709, 204576, 504237, and 203740 were grown in 4AA minimal medium supplemented with 10 μM ZnCl_2_. The presence of trace zinc decreased the ability of the supernatants to induce cGMP on T84 epithelial cells. Untx, untreated. Download FIG S4, EPS file, 1.6 MB. Copyright © 2020 Kiefer et al. 2020 Kiefer et al. This content is distributed under the terms of the Creative Commons Attribution 4.0 International license.

### Metallothionein detoxifies ST.

**FIG 6:**
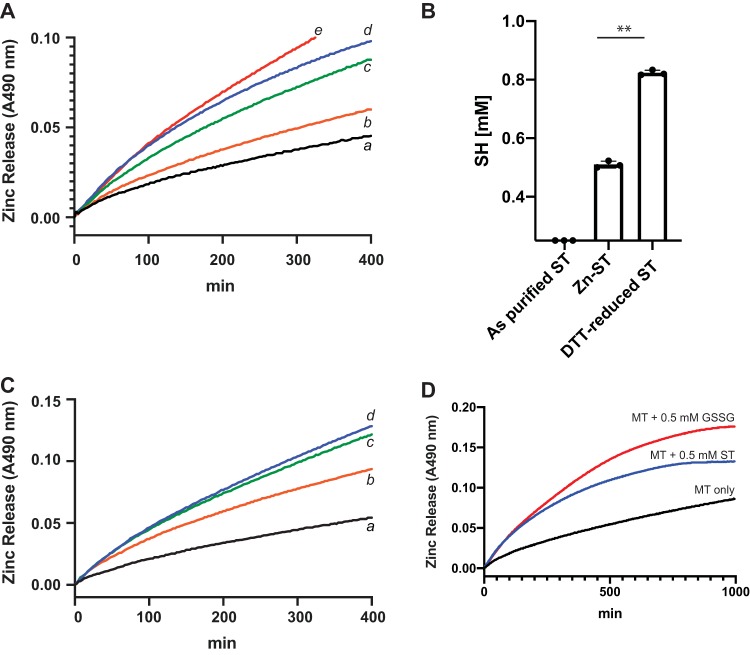
ST disulfide stress liberates zinc from metallothionein. (A) Metallothionein (4.17 μM, containing 0.1 mol zinc per mol protein) was incubated with excess disulfides and PAR at 37°C for up to 20 h to quantify zinc release from metallothionein. The rate of zinc release from metallothionein can be interpreted based on the slope of the absorbance at 490 nm. a, metallothionein only (black trace); b, metallothionein plus 40 μM ST (orange trace); c, metallothionein plus 200 μM ST (green trace); d, metallothionein plus 500 μM ST (blue trace); e, metallothionein plus 500 μM GSSG (red trace). (B) Purified ST was repurified using C_18_ chromatography under three conditions: (i) no treatment, (ii) after zinc reconstitution, and (iii) after DTT reduction. ST-containing fractions were pooled and analyzed for the presence of free sulfhydryl via DTNB (Ellman’s Reagent). **, *P* < 0.005. (C) ST increased the rate of zinc released from metallothionein in the presence of GSSG: a, metallothionein only (black trace); b, metallothionein plus 125 μM GSSG (orange trace); c, metallothionein plus 125 μM GSSG plus 125 μM ST (green trace); d, metallothionein plus 250 μM GSSG (blue trace). The data presented are representative of at least three replicates. (D) Disulfides GSSG (0.5 mM) and ST (0.5 mM) both exhausted zinc content from metallothionein from ST after 7 to 10 h of incubation. More zinc was quantifiable via PAR when GSSG was used as the oxidant than with ST, suggesting that the difference in PAR:Zn absorbance may be due to some ST-zinc equilibrium.

Metallothionein has the ability to bind 7 zinc atoms per protein, and it was previously shown that disulfide stress caused by excess oxidized glutathione (GSSG) or other disulfides oxidizes the zinc ligand-binding cysteines of metallothionein causing the release of zinc, as measured by PAR and Zincon (59) (see Fig. S5). Such a mechanism may require the disulfide oxidant to become reduced as a consequence of the interaction, and if that disulfide oxidant happened to be a bacterial toxin that requires intact disulfides for activity, then metallothionine could be used as a host detoxification system. Here, we determined the effect of disulfide stress, as represented by oxidized glutathione (GSSG) and disulfide ST, on the ability to liberate zinc from metallothionein. We used GSSG, as it was shown to release zinc from metallothionein only as a comparator to disulfide laden ST. We did not expect ST to bind zinc under these conditions, since reductants are not added to the incubations. We incubated metallothionein (Enzo; 4.17 μM, 0.12 μM Zn^2+^ per protein), GSSG or ST, and PAR together at 37°C and measured the kinetics of zinc release from metallothionine, as measured by the increase of the Zn:PAR_2_ complex at 490 nm. In this experimental setup, there was a slow release of zinc from metallothionine (Fig. 6A; a, black trace). However, addition of ST at 40 μM (b, orange trace), 200 μM (c, green trace), or 500 μM (d, blue trace) increased the rate of zinc release from metallothionine (Fig. 6A). The increased rate of zinc release from metallothionine suggests that ST is now in a mixed redox state with some oxidized and some reduced disulfides. Interestingly, 500 μM ST (Fig. 6A; d, blue trace) induced a similar rate of zinc release from metallothionine as 500 μM GSSG (Fig. 6A; e, red trace), suggesting that active ST ETEC infections add to luminal disulfide stress via modulation of the glutathione (GSH)/GSSG ratio. In an effort to explain how ST could be binding zinc, we measured the total thiols of ST repurified using a C_18_ chromatography under three conditions: (i) no treatment, (ii) after zinc reconstitution, and (iii) after DTT reduction. As shown in Fig. 6B, untreated disulfide-laden ST toxin did not contain measurable sulfhydryls following DTNB (Ellman’s reagent) quantification after repurification. DTT-reduced ST contained maximal sulfhydryls (0.8 mM for 2 mg ST peptide), and zinc-reconstituted ST contained roughly two-thirds of maximal sulfhydryls (0.52 mM for 2 mg ST peptide) (Fig. 6B). These data suggest that ST is binding zinc via two cysteine residues per peptide. In an effort to demonstrate ST induction of disulfide stress, we first determined the rate of zinc release from metallothionein at baseline (Fig. 6C; a, black trace), in the presence of 125 μM GSSG oxidant (Fig. 6C; b, red trace), and in the presence of 250 μM GSSG oxidant (Fig. 6C; d, blue trace). As shown, more zinc was released from metallothionein as the concentration of GSSG increased. Then, we mixed equal-molar ST (125 μM) and GSSG (125 μM) together and measured the rate of zinc release from metallothionein (Fig. 6C; c, green trace). As shown, the rate of zinc released from metallothionine in the reaction mixture containing equimolar distinct disulfides (GSSG plus ST) rescued the rate of zinc release from metallothionein when GSSG (250 μM) (Fig. 6C; d, blue trace) was used, suggesting that ST is contributing to disulfide-mediated release of zinc from metallothionein. Moreover, after 7 to 10 h of incubation between metallothionein and ST (Fig. 6D, blue trace), an apparent equilibrium (steady state) was reached, showing that ST-mediated zinc release from metallothionein is not as effective as GSSG-mediated zinc release from metallothionein. More steady-state zinc was quantifiable via PAR when GSSG was used as the oxidant compared to that with ST. It is currently unknown if one or more of the ST’s three disulfide bonds facilitates zinc release from metallothionine or if presentation of oxidative disulfide stressors is evolutionarily advantageous for ST^+^ ETEC proliferation in the host.

10.1128/mSphere.00146-20.5 FIG S5 Oxidized glutathione increases the rate of zinc release from metallothionein with respect to total glutathione concentration. Other disulfide stress, including secreted toxins, may modulate luminal redox potentials: PAR only (black trace), metallothionein only (red trace), metallothionein plus 0.125 mM GSSG (blue trace), metallothionein plus 0.25 mM GSSG (green trace), metallothionein plus 0.5 mM GSSG (purple trace), and metallothionein plus 1.0 mM GSSG (purple trace). Download FIG S5, EPS file, 1.6 MB. Copyright © 2020 Kiefer et al. 2020 Kiefer et al. This content is distributed under the terms of the Creative Commons Attribution 4.0 International license.

**FIG 7:**
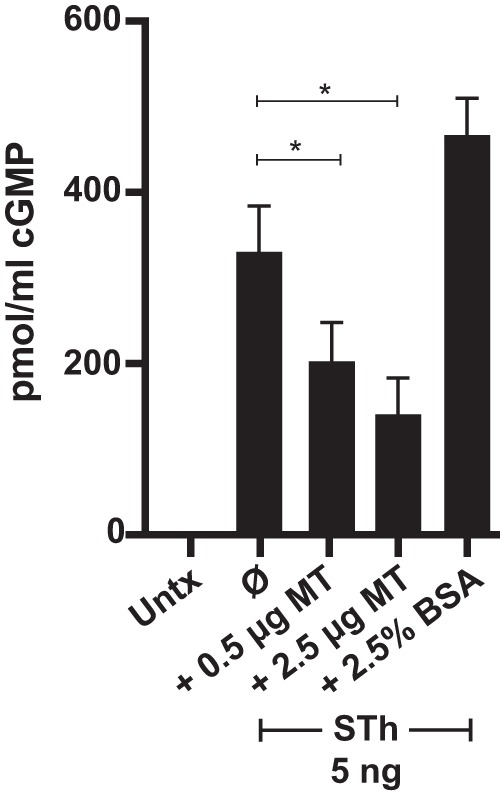
Metallothionein can detoxify ST. STh (5 ng) was preincubated with metallothionein (0.5 or 2.5 μg) or BSA (2.5%) for 24 h and then applied to T84 cells pretreated with zardaverine (20 μM) and vardenafil (50 μM) for 1 h. ST-mediated induction of cGMP was carried out for 2 h, and cGMP determination was carried out according to the manufacturer’s instructions. Significance was determined by the unpaired Mann-Whitney test. Values are means ± standard deviations versus ST alone. *, *P* < 0.05; Untx, untreated.

Since ST-mediated accumulation of cGMP in T84 cells requires ST to be in its disulfide state, we next incubated ST (5 ng) in the presence of increasing metallothionine (MT; 0.5 μg or 2.5 μg) for 24 h to determine if metallothionein can detoxify ST. We found that ST induction of cGMP in T84 cells was significantly decreased after preincubation with metallothionein (Fig. 7). We ruled out the potential for peptide interference with ST activity by preincubating ST with bovine serum albumin (BSA; 2.5%), which did not decrease ST activity (Fig. 7).

### ST modulates host transcription.

**FIG 8:**
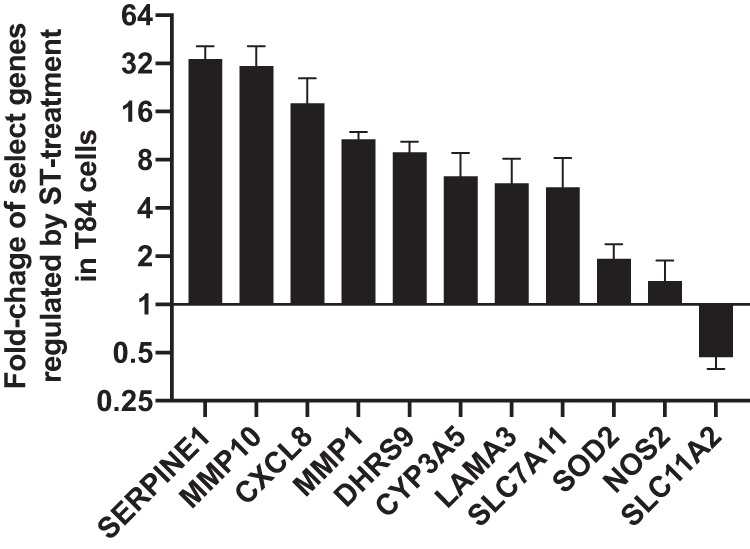
ST induces changes epithelial gene expression. STh (100 ng) was added to three confluent T84 epithelial monolayers for 3 h before RNA isolation. Another set of three monolaters were left untreated to serve as the control. RNA was isolated from all six monolayers and reverse transcribed into cDNA using iScript. qPCR was carried out using predesigned gene-specific primers from IDT, PrimeTime gene expression master mix, and approximately 10 ng of cDNA input from both ST-treated and untreated samples on a CFX Connect. *HPRT1* and *ACTB* were used as housekeeping genes, and the *y* axis represents the changes in transcript level of genes from T84 cells treated with ST compared to that in untreated cells.

To determine if ST alters epithelial cell transcriptional responses of genes involved in iron and zinc homeostasis or oxidative stress response pathways, we screened select genes previously identified to be regulated in intestinal epithelial cells by Streptomyces pilosus siderophore deferoxamine (60–63). Regulation of transcripts by ST was carried out by comparing the transcript level of target genes following 3 h of ST treatment on T84 cells to the transcript level of target genes in untreated T84 cells. ST treatment resulted in significant changes of transcript levels of the structural/epithelial remodeling genes laminin A3 (*LAMA3*), serpin E1 (*SERPINE1*), matrix metallopeptidase 1 (*MMP1*), matrix metallopeptidase 10 (*MMP10*), and iron and zinc transport gene divalent metal transporter 1 (*SLC11A2*), oxidative stress defense genes XCT (*SLC7A11*), cytochrome P450 3A5 (*CYP3A5*), nitric oxide synthase 2 (*NOS2*), superoxide dismutase 2 (*SOD2*), and dehydrogenase/reductase 9 (*DHRS9*), and interleukin 8 (IL-8) (*CXCL8*) (Fig. 8). These data demonstrate that ST may prime intestinal epithelial cells to change gene expression patterns to an inflammatory phenotype seen previously in human intestinal epithelial cells in response to iron chelation (60). More detailed studies are needed to determine if ST is directly (via binding to GC-C) or indirectly (by induction of cGMP) modulating gene expression or if chronic or acute ST (or iron/zinc ST) exposure accounts for some of the long-term morbidity seen in ST^+^ ETEC-mediated diarrheal disease.

**FIG 9:**
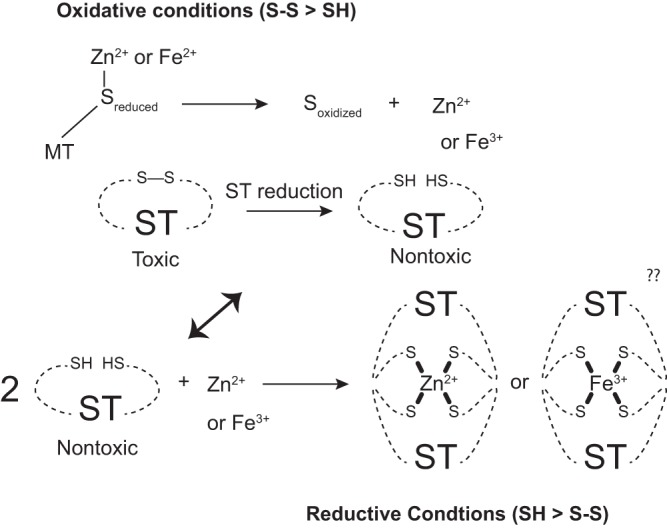
Working model on ST metal binding. Under oxidative conditions, the zinc-coordinating cysteines of metallothionein can be reduced to liberate free zinc, which then can bind to reduced ST upon a return to reducing conditions.

The intestinal lumen is considered anaerobic or microaerophilic at best, conditions that would significantly lower the reduction potential needed to reduce the disulfide bonds of ST. Based on the data presented here, we have developed a working model of metallothionein detoxification of ST (Fig. 9). Under disulfide stress, zinc is released from metallothionein, and upon return to reductive conditions, ST binds zinc in a 2 ST:1 zinc ratio. Potentially, mucosal iron-binding proteins such as lactoferrin may also modulate the toxicity of ST *in vivo*. Alternatively, luminal ST could be reduced *in situ* by an apical reductase, possibly dehydrogenase/reductase SDR family member 9, which is induced in T84 cells upon ST treatment (Fig. 8).

## DISCUSSION

It remains to be determined if transition metal binding provides ST ETEC a competitive advantage over LT ETEC or other enterics or if the host exploits transition metal binding by ST as a means of detoxification. Iron and zinc binding to ST could be beneficial to ETEC if it functioned as a siderophore or functioned to reduce luminal niche iron/zinc concentrations. Like iron, zinc is sequestered by proteins and through small molecules, and yersiniabactin of Yersinia pestis is both an iron and zinc siderophore (25). Recently, Haines et al. demonstrated that iron limitation stimulated *cfaA* promoter activity in ETEC H10407 through iron-sulfur cluster regulator protein IscR (26). Under the same conditions of iron limitation, LT secretion was inhibited, while transcription of *sta1* was roughly unchanged (26).

Indeed, according to recent global data, >65% of the wild ETEC isolates maintain ST, either alone or in combination with LT (64), and in a study surveying pediatric patients with environmental enteric dysfunction, ETEC was the only pathogen to induce elevated fecal calprotectin, a fecal maker of environmental enteropathy and a zinc- and iron-binding protein (65–67). In a recently developed ETEC colonization model using dietary zinc restriction, ETEC colonization was associated with higher intestinal titers of the inflammatory markers myeloperoxidase and lipocalin-2 (42). Moreover, lactoferrin inhibited adherence of ETEC to both human epithelial cells and to the intestinal mucosa of germfree mice (68). Host metal-binding proteins calprotectin, myeloperoxidase, and lactoferrin, typically derived from host neutrophils, have become fecal markers of ETEC infection, and recent studies have shown that these biomarkers can be used to assess the burden of enteric infections (42, 65, 69–71).

Historically, ETEC has not been associated with inflammation, but epithelial cell phenotypes during and following infection suggest that inflammation occurs (42, 65). Such inflammatory conditions lead to imbalances in luminal and cellular transition metal pools (72, 73). Indeed, imbalances in transition metals using siderophores can also lead to the induction of inflammatory genes (60). The role for transition metals in the regulation of ETEC virulence is supported with IscR-mediated biofilm formation in E. coli and IscR-mediated virulence in Shigella flexneri, Erwinia chrysanthemi, and Pseudomonas aeruginosa (28–30). An *fnr* mutant of uropathogenic E. coli strain CFT073 was highly attenuated in the mouse model of human urinary tract infection and showed severe defects in bladder and kidney adherence (74). In regard to zinc, maintenance of high-affinity zinc uptake systems, including *znuABC*, is advantageous for adherence to Caco-2 cells. Indeed, *znuA* was strongly induced in E. coli O157:H7 adherent to Caco-2 cells, and a *znuA*-deficient mutant was unable to compete with wild-type strains in adherence (75).

ST enterotoxin has six cysteines, a versatile amino acid capable of forming disulfide bonds or coordinating transition metals. The presence of six cysteines may signify multifunctionality, and the discovery of novel isoforms and metal binding may not seem so surprising. For example, uroguanylin, the host natriuretic peptide that ST mimics, has only four cysteine residues and has at least two different topoisomers, each having a different affinity for the GC-C receptor (14). It is plausible to envision ST having more topoisomers depending on when the last disulfide bond is formed. Improperly folded ST is further processed by DsbC before leaving the periplasm of ETEC (76), and it is likely that multiple forms escape the periplasm, since we and others have seen ST toxin activity in up to five discrete peaks after reverse-phase C_18_ chromatography. Alternatively, *in situ*, as quickly as ETEC produces ST disulfide, it may bind to the GC-C receptor or begin to become reduced, one disulfide at a time, creating a pool of redox-active STs that have various affinities for the GC-C receptor. In such a scenario, ETEC would maintain a threshold rate of ST disulfide production in order to elicit disease.

Identification of iron- and zinc-binding abilities for ST represents a more complex emerging story regarding the host-pathogen interface. Potentially, ST can exist in both forms at the same time or be interconverted between a disulfide-rich toxin and a metal binder under different environmental conditions, including redox potentials, different phases of infection, or depending on the particular host. It should be noted that in experimental animal models of enterotoxin activity, ST induces fluid movement more quickly than LT. Zinc and iron binding to ST may be a function of environmental conditions, since *in vitro* ST iron reconstitution only occurs anaerobically and ST zinc reconstitution occurs aerobically but only under reducing conditions. More studies will be needed to determine if one of these metals binds preferentially and to determine the coordination of the metal centers and oxidation states. Recently, zinc hook peptides have been engineered using a peptide sequence of Rad50 to generate highly stable Zn(II) complexes (77). The 14-amino-acid sequence of Rad50 shares similarity to the amino acid sequence of ST, and the stoichiometry of 2 ST:1 zinc is supported by two monomers of Rad50 coalescing around one ZnS_4_ cross-link between each zinc hook domain (77). Multifunctionality partially explains the evolutionary pressure as to why some ETEC strains expend the metabolic energy to maintain and produce ST regardless of LT, such as H01407.

The cysteines of ST bridge three disulfide bonds, which imparts it with heat and conformational stability. Proper configuration of disulfide bond chemistry is mediated by the thiol/disulfide oxidoreductase DsbA (49). *In vitro*, reduction of disulfides is accomplished using beta-mercaptoethanol (β-ME) or DTT, and previously, thiols have been used to inactivate ST, as measured by the suckling mouse model (78, 79). *In vivo*, disulfide reduction is carried out through enzymatic systems, including thioredoxins or glutaredoxins poised with redox potentials of NADPH or GSH, respectively. However, *in situ*, such as the lumen of the intestines, redox conditions of the microbiome-colonized intestinal lumen are not fully understood, and it remains to be seen whether redox active metabolites from host diet and metabolism (luminal glutathione [GSH]) or microbiome-derived fermentation by-products (H_2_S) are able to modulate the activity of ST. Luminal total thiol concentrations have been estimated to be 100 to 200 μM in the jejunum of the rat intestines, with cysteine as the dominant mucosally derived thiol (80). GSH has the ability to reduce disulfides (81), scavenge metals (82), and maintain mucous fluidity (83). GSH deficiency in mice leads to degeneration of the jejunum and colon (84), and this could partially explain blunting of villi. Basal biliary excretion of GSH has been estimated to be approximately 14.8 nmol/min/100 g body weight of Sprague-Dawley rats (85).

Zinc is intimately tied to luminal GSH pools and oxidative stress defense. Zinc is tightly bound to proteins, and the regulation of zinc distribution and mobilization is still not fully understood. Metallothionine (MT) is a low-molecular-weight host protein that can bind up to 7 zinc atoms with high affinity, and the low redox potential of MT (<−366 mV) allows effective oxidation by mild cellular oxidants, including disulfides such as GSSG (59). Interestingly, DsbA, which coordinates the disulfide bond coordination of ST, reacts stoichiometrically with metallothionein to release zinc (59). One potential mechanism through which epithelial cells could detoxify ST is by increasing metallothionein concentrations. It has long been known that zinc supplementation (i) reduces the severity and duration of diarrheal diseases and (ii) induces intestinal metallothionein concentrations (86). Research in our lab is currently investigating the potential of the disulfide/sulfhydryl pair in modulating infection.

The presence of luminal host natriuretic peptides, guanylin and uroguanylin, may hinder the search for redox forms of ST from intestinal lavages, and it remains to be determined if guanylin and uroguanylin bind transition metals. Supporting our findings, investigators recently identified BEST4^+^OTOP2^+^ colonic epithelial cells that secrete uroguanylin and guanylin, express proteins that regulate luminal pH, and express genes of the metallothionein family, contributing to free radical defense and metal ion storage and transport (34).

## MATERIALS AND METHODS

### Media and strains.

We used the following ETEC isolates: STp-overexpressing B41, 214-4 (kindly provided by Myron Levine, University of Maryland School of Medicine), STh-overexpressing strain 9115 (kindly provided by Weiping Zhang, University of Illinois at Urbana—Champaign), and clinical ST-only ETEC isolates 504239, 504838, 300709, 204576, 504237, and 203740 (3). For general maintenance, ETEC strains were streaked from glycerol stocks onto LB agar plates (containing 100 μg/ml ampicillin, when needed). Experiments were carried out in 4-amino-acid (4AA) minimal medium, as previously described by Alderete and Robertson (87). In brief, 4AA minimal medium components include: aspartic acid (6.61 mM), proline (12.3 mM), alanine (4.5 mM), serine (6.3 mM), NaCl (42.8 mM), K_2_PO_4_ (50.0 mM); NH_4_Cl (18.7 mM), Na_2_SO_4_ (1.4 mM), Tricine (5.6 mM), sodium lactate (0.05%), MgCl_2_ (246.0 μM), MnCl_2_ (25.0 μM), and FeCl_3_ (31.0 μM). Single colonies of ETEC were picked from the LB agar and first inoculated into LB overnight at 37°C and 250 rpm. LB broth cultures were then subcultured 1:100 in 10.0 ml of 4AA minimal medium in 100-ml Erlenmeyer flasks (1:10, air/liquid) for 6 h at 37°C and 250 rpm (adaptation phase) before being subcultured again 1:100 into 25.0 ml of fresh 4AA minimal medium in 250-ml Erlenmeyer flasks overnight at 37°C and 250 rpm. After overnight incubation, ETEC cultures were processed, optical densities were determined, and supernatants were collected and sterilized using 0.2-μm membrane filters. Supernatants were assayed for total protein content using the Pierce bicinchoninic acid (BCA) protein assay kit, and UV-visible spectra were recorded using an Epoch2 spectrophotometer (Bio-Tek) equipped with a cuvette chamber. Supernatants were stored at −20°C until they were assayed for ST activity (see below). In ST^+^ ETEC growth experiments requiring different concentrations of iron, FeCl_3_ was titrated into the 4AA minimal medium from 0, 2.5, 5.0, 30.0, or 60 μM while maintaining the concentrations of other medium components. In ST^+^ ETEC growth experiments with replete zinc, ZnSO_4_ (10 μM) was added to the 4AA minimal medium. Secreted protein concentrations were quantified (Pierce BCA protein assay kit) from supernatants grown at different iron or zinc concentrations, and the same mass of total secreted protein (5.0 μg) was applied to individual wells of flat-bottom culture plates containing confluent T84 epithelial cells, which were assayed for cGMP as described below.

### Purification of ST.

The heat-stable enterotoxin STh was purified from E. coli strain 9115, and the heat-stable enterotoxin STp was purified from wild-type E. coli strain B41 (88, 89). Large-scale (9.9 liter) fermentation of STh from ETEC strain 9115 or B41 was according to the culturing strategy described above, except seed cultures were scaled for a final volume of 10 liters. ETEC 9115 was grown in a 20-liter carboy fitted with a gas sparger (2308-A04-06-A00-2-AB; Mott Corporation, Farmington, CT) connected to medical-grade nitrogen (flow rate at 3 liters/min) and medical-grade oxygen (flow rate at 0.3 liters/min) overnight at 37°C with a stir bar set to maintain agitation at 200 rpm. The following morning, the cells were pelleted by centrifugation at 8,000 rpm for 10 min in a Sorvall Lynx 6000, and large-molecular-weight proteins were removed via tangential flow filtration by passing the ST-containing supernatant through a Pellicon 3 Ultracel 10 kDa membrane (P3C010C01; EMD Millipore). The low-molecular-weight ST-containing filtrate was applied to 500 g of Amberlite XAD-2 (10357; Sigma-Aldrich) hydrophobic-interacting resin, and ST-containing material was eluted by adding methanol (99.9%) and trifluoroacetic acid (0.01%) followed by a second elution using methanol (79.9%), trifluoroacetic acid (0.01%), and water (20%). The ST-containing eluate was concentrated 5-fold, and methanol was removed using a using a Rotavapor R-210 (Buchi) before adding phosphate buffer and sodium chloride and adjusting the pH to 7.4. The ST-containing XAD-2 eluate was then concentrated using the Rotavapor R-210 until 10 to 12 ml remained. A 5.0-ml aliquot of concentrated XAD-2 eluate was applied to a Bio-Gel P6 gel filtration column (75 cm by 2.5 cm) preequilibrated in 20 mM Tris, 0.2 M NaCl, pH 7.4, and attached to a Bio-Rad DuoFlow HPLC system. Concentrated XAD-2 eluate was collected up to 600 ml, and individual 3-ml fractions were assessed for ST in the T84 assay described below. Pooled ST-containing fractions (∼80 ml) were concentrated with a Rotavapor R-210. Post-P6 ST eluate (5.0 ml) was then applied to a Waters Spherisorb S5 ODS2 C_18_ column, preequilibrated with 50 mM ammonium acetate, pH 5.4, attached to the Bio-Rad DuoFlow HPLC system. ST was eluted from the C_18_ column using 50 mM ammonium acetate, pH 5.4, dissolved in methanol. Individual 3-ml fractions were assayed for ST activity, and fractions containing ST were pooled. STh eluted as a doublet over 5 fractions (15 ml) and STp eluted as a singlet over 3 fractions (9 ml) at ∼50% methanol. Phosphate buffer and sodium chloride were added to the pooled purified ST, and the pH was adjusted to 7.4. Finally, methanol was removed from purified ST preparations using the Rotavapor R-210. Purified ST was washed with >100 ml type I water, concentrated to 1.0 mg/ml, and stored at −20°C until needed. STh purified using this methodology has been deposited into BEI Resources under the catalogue numbers NR-50760, NR-50761, NR-50762, NR-50763, NR-50764, and NR-50765.

### T84 cell culture and cGMP assay.

Human T84 colonic epithelial cells were purchased from American Type Culture Collection (catalog number CCL-248). T84 cells were cultured in 1:1 Dulbecco’s modified Eagle’s medium and Ham’s nutrient mixture F-12 (DMEM-F-12) containing 2.5 mM l-glutamine, 15 mM HEPES, 0.5 mM sodium pyruvate, and supplemented with 5% fetal bovine serum (FBS). All cell cultures were supplemented with antibiotic-antimycotic (Gibco). Cells were maintained in T-75 culture flasks incubated at 37°C and 5% CO_2_ humidified air. Cells at passages 2 through 11 were used for all experiments. Confluent T84 cells were harvested from T-75 culture flasks using 0.25% trypsin and resuspended in DMEM-F-12 medium. T84 cells were seeded into 24-well flat-bottom cell culture plates (CLS3526; Corning Costar) at a density of 5 × 10^5^ cells/cm and grown to confluence. Intracellular cGMP was determined as previously described (90). Briefly, T84 cells at confluence were incubated in DMEM-F-12 containing 1% FBS with 20 μM zardaverine (Z3003; Sigma-Aldrich) and 50 μM *N*-desethyl vardenafil (448184-46-1; Cayman Chemicals) at 37°C and 5% CO_2_ (91–93). Following a 1-h preincubation with phosphodiesterase inhibitors, ST or ST^+^ ETEC culture supernatants were applied to the monolayers. After 2 h of treatment, T84 monolayers were washed three times with cold phosphate-buffered saline (PBS), pH 7.4. The cells were lysed, and the intracellular cGMP content was determined using a cGMP parameter assay kit (SKGE003; R&D Systems) according to the manufacturer’s instructions.

### Iron binding assays.

Purified STh and STp (100 μM each) were incubated aerobically (atmospheric levels) or anaerobically (in argon-purged vials) for 30 min at 37°C with ferrous ammonium sulfate (400 μM) and DTT (2 mM) (94). After incubation, the samples were repurified using a HiTrap desalting column (GE Healthcare) attached to a Bio-Rad DuoFlow HPLC systems. Iron-reconstituted ST-containing fractions were immediately transferred to argon-purged anaerobic vials. UV-Visible spectra of the reconstituted samples were recorded using an Epoch2 spectrophotometer. Iron contents of STh, STp, and C_18_ elution fractions were determined using the iron quantification reagent Ferrozine (ε_562nm_, 27.9 mM^−1^ cm^−1^) in the presence of excess cysteine, as performed previously (51).

### Zinc binding assays.

For zinc binding experiments, STp or STh (10 μM or 40 μM) was incubated aerobically with zinc sulfate (up to 20 μM) and DTT (2 mM) for 20 min at room temperature. Then, 4-(2-pyridylazo)resorcinol (PAR) (100 μM) was added to the reactions and incubated for an additional 5 min at room temperature. Zinc binding to STp and STh was determined by the absorbance of the Zn:PAR_2_ complex at 490 nm (95, 96) on a BioTek Epoch 2 plate reader. When releasing zinc from Zn-STp or Zn-STh, H_2_O_2_ (4.0 mM) was added in the presence of PAR and allowed to incubate overnight at room temperature. Zinc release from STp or STh was quantified by the appearance of the Zn:PAR_2_ complex peak at 490 nm.

### Zinc release from metallothionein.

Metallothionein MT1A was purchased from Enzo (catalog number ALX-202-070-C500) and reconstituted to 1 mg/ml (140 μM protein). The fraction of zinc binding to metallothionein was determined by PAR quantification, using a zinc standard curve from 0 to 20 μM. In all zinc release experiments, metallothionein (4.2 μM) was incubated with PAR (100 μM) and ST, oxidized glutathione (GSSG), or both at different concentrations. The contents of the reaction mixtures were placed onto a 96-well plate, overlaid with 50 μl mineral oil, and placed into an Epoch 2 spectrophotometer with the temperature fixed to 37°C. Absorbances at 490 nm and 450 nm (background reading) were monitored every 2.5 min for 20 h. Zinc release from PAR was carried out in triplicates for each experimental condition.

### qPCR.

T84 epithelial cells were grown to confluence on 24-well tissue culture-treated plates. ST (100 ng) was added to the cells for 2 h followed by RNA extraction with Qiagen RNeasy. RNA isolated from untreated T84 monolayers served as the control. RNA was quantified using a NanoDrop C, and 1.0 μg RNA was added to tubes for reverse transcription with and without iScript (Bio-Rad). qPCR was carried out using predesigned gene-specific primers from IDT (Table 1) and PrimeTime gene expression master mix on a CFX Connect (Bio-Rad). We did not see genomic DNA contamination in our RNA samples, as tested by performing qPCRs on RNA samples without iScript in the reverse transcription reaction. Transcript levels were calibrated to housekeeping genes *HPRT1* and *ACTB*. Changes in mRNA expression were determined using the comparative threshold cycle (*C_T_*) method (97).

**TABLE 1:** qPCR primers used for the interrogation T84 gene expression following 3 h of 100-ng ST treatment

Primer	Vendor catalog no.^*a*^	Sequence (5′→3′)^*b*^
HPRT1 p1	Hs.PT.58v.45621572	TTGTTGTAGGATATGCCCTTGA
HPRT1 p2		GCGATGTCAATAGGACTCCAG
HPRT1 probe		/56-FAM/AGCCTAAGA/ZEN/TGAGAGTTCAAGTTGAGTTTGG/3IABKFQ/
ACTB p1	Hs.PT.39a.22214847	ACAGAGCCTCGCCTTTG
ACTB p2		CCTTGCACATGCCGGAG
SLC7A11 p1	Hs.PT.58.20878688	TCATTGGAGCAGGAATCTTCATC
SLC7A11 p2		GTTCCCAATTCAGCATAAGACAA
SLC7A11 probe		/56-FAM/TGTTCTGGA/ZEN/GCACGCCCTTAGG/31ABkFQ/
MMP1 p1	Hs.PT.58.38692586	GGACGCATTCAGAAGGAACA
MMP1 p2		GCAACTTCAGCTTTCAGTTCA
MMP1 probe		/56-FAM/TCCGTGTAG/ZEN/CACATTCTGTCCCTG/31ABkFQ/
CYP3A5 p1	Hs.PT.58.2827789	CCCATTCCGTCACCATGT
CYP3A5 p2		TCTCTTCCATTCTTCATCCTCAG
CYP3A5 probe		/56-FAM/CCTCCTGCT/ZEN/GTCCTACAGTCACAAC/31ABkFQ/
NOS2 p1	Hs.PT.58.14740388	CACCATCCTCTTTGCGACA
NOS2 p2		GCAGCTCAGCCTGTACT
NOS2 probe		/56-FAM/TATTCAGCT/ZEN/GTGCCTTCAACCCCA/31ABkFQ/
SERPINE1 p1	Hs.PT.58.3938488.g	TGACAACAGGAGGAGAAACC
SERPINE1 p2		GAGCTCCTTGTACAGATGCC
SERPINE1 probe		/56-FAM/TGCCCTTGT/ZEN/CATCAATCTTGAATCCCA/31ABkFQ/
LAMA3 p1	Hs.PT.58.836388	TTACCACCTACTGACCACCT
LAMA3 p2		GTAACCATCTTCCAGAGTGACC
LAMA3 probe		/56-FAM/TCAGACCTT/ZEN/TCAACCCAGTGGCAT/31ABkFQ/
DHRS9 p1	Hs.PT.58.14588753	GGAAGACACAGCAGATAAGCA
DHRS9 p2		AGTCCACAGAAAACCACAGAG
DHRS9 probe		/56-FAM/AACTCAAGC/ZEN/AACCAGGACACCATCT/3IABKFQ/
SOD2 p1	Hs.PT.58.25533008	GACAAACCTCAGCCCTAACG
SOD2 p2		CGTCAGCTTCTCCTTAAACTTG
SOD2 probe		/56-FAM/CTTCCAGCA/ZEN/ACTCCCCTTTGGGT/3IABKFQ/
MMP10 p1	Hs.PT.58.38586852	GGAGACTTTTACTCTTTTGATGGC
MMP10 p2		AGCAACGAGGAATAAATTGGTG
MMP10 probe		/56-FAM/ACAGTTTGG/ZEN/CTCATGCCTACCCA/31ABkFQ/
SLC11A2 p1	Hs.PT.58.23097204	TTGCGGAGCTGGTAAGAATC
SLC11A2 p2		CCCATGATCTCCAGAAACACT
SLC11A2 probe		/56-FAM/TGGTGGATA/ZEN/CCTGAGTGGCTGAGT/3IABKFQ/
CXCL8 p1	Hs.PT.58.39926886.g	GAGACAGCAGAGCACACAAG
CXCL8 p2		CTTCACACAGAGCTGCAGAA
CXCL8 probe		/56-FAM/AGGACAAGA/ZEN/GCCAGGAAGAAACCAC/31ABkFQ/

a All primers are from IDT, Inc., and are human-specific.

b FAM, 6-carboxyfluorescein.

### Patent mouse model.

Animal studies were approved by the Tulane University Institutional Animal Care and Use committee. Adult patent mouse assays were conducted on female BALB/c mice from Charles River Laboratories. The mice were given saline, 25 μg ST, or 25 μg ST-iron via gastric lavage using a bent 20-gauge feeding needle. Following toxin administration, adult mice were incubated for 30 min at room temperature. The mice were sacrificed by CO_2_ inhalation, the entire intestine from the duodenum to the rectum was removed, and the gut-to-carcass ratio was determined. The mice were maintained on standard laboratory diet, but food was denied for 18 h prior to ST gavage, while water was allowed *ad libitum*.

### Statistical analysis.

Statistical analysis was performed using Prism 8 software (GraphPad, Inc.). In experiments containing two groups, statistical analysis was performed using unpaired *t* tests. In experiments with more than two groups, statistical analysis was performed using unpaired one-way analysis of variance (ANOVA), followed by Tukey’s or Bonferroni’s *post hoc* analysis as appropriate; a *P* value of <0.05 was considered significant.
